# American Medical Editors

**Published:** 1883-07-20

**Authors:** 


### *AMERICAN MEDICAL EDITORS.*

The Association of American Medical Editors was held on June 5th, inst., at Cleveland, Ohio.

An interesting address was made by Dr. N. G. Davis, the president, on the present status and future tendencies of the medical profession, and of medical journalism.

Dr. Marcy made an address on "Journalism devoted to the protection and concentration of Medical and Surgical Science in Special Departments."

Dr. Leartus Conner, of Detroit, was elected President, and Dr. Thomas Gallagher, of Pittsburg, Vice-President.

Dr. J. V. Shoemaker, Secretary, for the ensuing year.

